# Cognitive enhancement effects of stimulants: a randomized controlled trial testing methylphenidate, modafinil, and caffeine

**DOI:** 10.1007/s00213-020-05691-w

**Published:** 2020-11-17

**Authors:** Dimitris Repantis, Leonore Bovy, Kathrin Ohla, Simone Kühn, Martin Dresler

**Affiliations:** 1 Department of Psychiatry and Psychotherapy, Campus Benjamin Franklin, Charité – Universitätsmedizin Berlin, corporate member of Freie Universität Berlin, Humboldt-Universität zu Berlin, and Berlin Institute of Health, Hindenburgdamm 30, 12203 Berlin, Germany; 2 Lise Meitner Group for Environmental Neuroscience, Max Planck Institute for Human Development, Berlin, Germany; 3 Donders Institute for Brain, Cognition and Behaviour, Radboud University Medical Center, Nijmegen, The Netherlands; 4 Institute of Neuroscience and Medicine (INM-3), Jülich Research Centre, Jülich, Germany; 5 Department of Psychiatry and Psychotherapy, University Medical Center Hamburg-Eppendorf (UKE), Hamburg, Germany

**Keywords:** Neuroenhancement, Cognitive enhancement, Methylphenidate, Modafinil, Caffeine

## Abstract

**Rational:**

At all times humans have made attempts to improve their cognitive abilities by different means, among others, with the use of stimulants. Widely available stimulants such as caffeine, but also prescription substances such as methylphenidate and modafinil, are being used by healthy individuals to enhance cognitive performance.

**Objectives:**

There is a lack of knowledge on the effects of prescription stimulants when taken by healthy individuals (as compared with patients) and especially on the effects of different substances across different cognitive domains.

**Methods:**

We conducted a pilot study with three arms in which male participants received placebo and one of three stimulants (caffeine, methylphenidate, modafinil) and assessed cognitive performance with a test battery that captures various cognitive domains.

**Results:**

Our study showed some moderate effects of the three stimulants tested. Methylphenidate had positive effects on self-reported fatigue as well as on declarative memory 24 hours after learning; caffeine had a positive effect on sustained attention; there was no significant effect of modafinil in any of the instruments of our test battery. All stimulants were well tolerated, and no trade-off negative effects on other cognitive domains were found.

**Conclusions:**

The few observed significant positive effects of the tested stimulants were domain-specific and of rather low magnitude. The results can inform the use of stimulants for cognitive enhancement purposes as well as direct further research to investigate the effects of stimulants on specific cognitive domains that seem most promising, possibly by using tasks that are more demanding.

**Supplementary Information:**

The online version contains supplementary material available at 10.1007/s00213-020-05691-w.

## Introduction

At all times, humans have made attempts to improve their cognitive abilities (Dresler et al. 2018). Several means of enhancing cognition are every day practice, such as the use of caffeine in the form of caffeine-containing beverages (Dresler et al. 2013). Other interventions however raise ethical and legal concerns such as the use of prescription medications, a phenomenon that has been termed pharmaceutical neuroenhancement or cognitive enhancement (Farah 2015; Maier et al. 2015a; Maier et al. 2015b; Repantis et al. 2010). There are a number of substances that are presumably being used for this purpose including, among others, prescription stimulants, for example methylphenidate (MPH), a catecholamine reuptake inhibitor that is mainly used in the treatment of attention-deficit/hyperactivity disorder. Although there is some laboratory and anecdotal evidence available showing a positive effect of MPH on the cognition of healthy individuals, there are also studies showing no or even detrimental effects (Repantis et al. 2010). Modafinil (MOD) is a non-amphetamine stimulant that is also being used as a neuroenhancing substance. Due to its wakefulness promoting properties, it is used for the treatment of narcolepsy. Its mode of action is less clear, but it seems that it increases extracellular catecholamine levels and indirectly activates the hypocretinergic system (Minzenberg and Carter 2008). A systematic review of studies on sleep-deprived healthy individuals suggests that MOD maintains wakefulness and to some extent enhances cognitive function to a higher degree than placebo (Repantis et al. 2010). Studies on non-sleep-deprived individuals paint however a more complex picture, with, among others, several studies showing null effects (Battleday and Brem 2015; Repantis et al. 2010). Epidemiological data suggest a widespread use of prescription stimulants (primarily amphetamines, but also MPH) for cognitive enhancement purposes, albeit with great variation in prevalence based on the country and population studied. In a systematic review of 21 studies examining the lifetime prevalence of non-prescribed stimulant use there was a range from 5 to 9% in grade school- and high school-age children and 5 to 35% in college-age individuals. This use was mostly for cognitive enhancement, but also to “get high” or experiment (Wilens et al. 2008). Studies in Germany show a lifetime use prevalence for cognitive enhancement between 1.3 and 5% (Franke et al. 2014; Sattler 2016). In a recent representative study of the US adult population a last year prevalence of non-medical use of 2.1% was found, whereas cognitive enhancement was with 78% the most commonly reported reason for non-medical use (Compton et al. 2018). Nevertheless this is surprising given the paucity of data showing actual enhancing effects in well-controlled studies on non-sleep-deprived healthy individuals. This is however interesting for several reasons. Finally, for a large amount of people, caffeine (CAF) seems to be the most obvious option for a cognitive-enhancing substance. In comparison to prescription stimulants, CAF is readily available in beverages but also in pills that are also being used for enhancement purposes (Franke et al. 2014). From a psychopharmacological perspective, CAF is interesting as a cognitive enhancer since it has a different mode of action with its effects being mediated through non-selective antagonistic effects on A_1_ and A_2A_ adenosine receptors, whereas MPH and MOD affect primarily dopaminergic and noradrenergic neurotransmission (Luethi et al. 2018; Wood et al. 2014). Therefore, there is a need for effectiveness studies to juxtapose the effects of prescription stimulants with those of caffeine in the same test battery. In order to address this question, we performed a placebo-controlled study with three arms testing three different stimulants (MPH, MOD, CAF) to explore the effects of several different popular stimulants on cognition. Each participant received placebo and one stimulant on two different experimental days and thus served as its own control. We hypothesized different effects in different areas of cognition for each stimulant, without however predicting in advance which domain would be most affected by which stimulant. We used a task battery that addresses diverse cognitive capacities (perceptual speed, working memory, episodic memory) and in diverse stimulus domains (numerical, verbal, figural-spatial) in order to tackle the different effects while also establishing commensurability in trade-offs between different domains.

## Experimental procedures

**Fig. 1:**
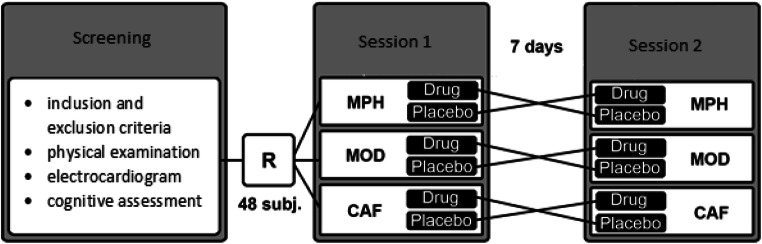
Baseline characteristics of the three groups and study design. After screening, 48 subjects were randomized (R) in a double-blind fashion to one of the three arms (MPH: methylphenidate, MOD: modafinil or CAF: caffeine)

Forty-eight healthy, right-handed male volunteers (age range = 21–36 years, *M* = 26. 27, *SD* = 3.47; Fig. 1) were recruited through internet advertisement and were compensated for their participation. Women were excluded due to the proposed interaction of the female hormone cycle and performance in cognitive tests (Cahill 2006). All subjects were screened for the presence of psychiatric disorders using the Mini-International Neuropsychiatric Interview (Sheehan et al. 1998). All participants were without current and past medical, neurological, and psychiatric disorders and without known psychiatric disorders in their family history. All denied use of prescription medications, nicotine, or illicit substances. Participants with excessive alcohol drinking or history of drug abuse were also excluded. In order to avoid recruiting participants that might nevertheless use drugs of abuse, participants were told that a drug urine screening might be performed at any point during the study. Moreover, every participant was screened by a psychiatrist at every visit before given the study medication. Habitual consumption of small quantities of caffeinated drinks was allowed, whereas regular as well as excessive consumption (> 4 cups/day) was not allowed. General intelligence was assessed using the Lern- und Gedächtnistest 3 (Bäumler 1974), the more abstract and culture-free intelligence measurement Culture Fair Intelligence Test (CFT-20R) (Weiß et al. 2006) and the Mehrfach Wortschatz Test (MWT-B) for crystallized intelligence (Lehrl 2005). Participants in the three arms did not differ in any of the baseline characteristics or intelligence scores. The study was registered in clinicaltrials.gov (No. NCT02071615), was performed under a protocol approved by the independent Berlin State Ethics Committee (LAGeSo Berlin, Germany; 13/0138-EK12), and was conducted according to the code of ethics on human experimentation established by the Declaration of Helsinki (1964) and its amendments. Written informed consent was obtained from all participants after a full explanation of study procedures, adverse reactions to drug treatment, legal rights and responsibilities, expected benefits of a general scientific nature, and their right for voluntary termination without penalty or censure.

### Procedure

The study was conducted according to a randomized, double-blind, placebo-controlled, within-subject design and had three arms testing three different stimulants vs. placebo (Fig. 1). Allocation to one of the three arms was double-blinded and each volunteer participated in only one arm and received only one stimulant. Each volunteer participated in two sessions separated by approximately one week (but not less than four days). Every session was scheduled at the same time in the early afternoon. Participants received placebo (microcrystalline cellulose) and a single oral dose of one of three stimulants: 20 mg of immediate-release methylphenidate or 200 mg of modafinil or 200 mg of caffeine. We chose these doses because it has been shown for MPH and MOD that they lead to an equivalent dopamine transporter occupancy (Volkow et al. 2009; Volkow et al. 1998) and because in clinical practice and previous studies they have been shown to be equipotent (Franke et al. 2017; Repantis et al. 2010; Theunissen et al. 2009). Participants were not allowed to use alcohol on the day prior to a session and were requested to arrive at experimental sessions well rested. Participants completed a memory task while in an MRI scanner (MRI data are not reported in the current study) and a number of further cognitive tests outside the scanner. The start of the cognitive testing was 90 min after substance ingestion. This timeframe was chosen in order to ensure that all three stimulants had approximately reached their peak concentration in blood during testing. Heart rate and blood pressure as well as the presence of side effects were assessed regularly and before discharge. All participants were contacted by telephone 24 h after the session in order to assess late occurring side effects, such as sleep disturbances and to conduct a pre-announced late recall test of the memory tasks.

### Measures

A number of cognitive tests were applied, chosen to test a wide and differential selection of cognitive functions. The tests were conducted according to fixed processing times for each test and without breaks in between. Parallel versions of the cognitive tasks where used. The allocation of each participant in one of the two arms (placebo/stimulants) was random; therefore the order of the parallel versions was not randomized.

In a *declarative memory task*, participants learned an array of 72 everyday language German words as well as the sequence in which they were presented. All words were common nouns; encoding difficulty was matched between lists and tested in pilot trials. Word lists counting about 70 words prevent ceiling effects and therefore seem to be adequate for healthy young subjects (Riedel and Blokland 2015). The words were presented in 12 blocks with six words each. Each word was presented for 2000 ms with a jittered inter stimulus interval of 2–5 s. Blocks were interspersed with fixation periods. Outside the scanner, approximately 20 min after learning the words, an early recall of the learned words was performed.

Then *logical reasoning* was tested using the BOMAT (Bochum matrices-advanced test; (Hossiep et al. 2001). In this test, geometrical figures have to be selected according to logical reasoning from the patterns of other geometrical figures in 5 x 3 matrices. Participants were instructed to complete as many matrices as possible within 10 min. The outcome measure was the percentage of correctly finished items within the sum of all answered items.

To measure the *speed of information processing*, a trail making test was conducted (ZVT–“Zahlenverbindungstest,” i.e., number connection test, Oswald and Roth 1987). It requires participants to connect numbers from one to 90 in ascending order by drawing a line. Participants are asked to connect the numbers as fast as possible; the average time across two trials was taken as outcome measure.

As a test of *working memory*, we used the Reverse Digit Span Test (Richardson 2007). Participants had to recall and write down in reverse order digits presented on a screen. Three to a maximum of ten consecutive digits and two trials per span were used. The longest span of digits repeated correctly (“BackSpan”) was the outcome measure.

*Creativity* and divergent thinking were measured with the alternate uses task: participants were asked to report as many unusual and creative uses for an object cue as possible in 1 min. Three rounds were performed, and the outcome measure here was the mean number of uses reported (Guilford 1967).

We also measured *implicit and explicit verbal memory* capacity and precision with a false memory test (inspired from Roediger and McDermott 1995). Participants heard through headphones 75 words sorted in five sets of 15 words. Every set contained several groups of semantically similar words. The words of each of these sets could all be associated with one critical lure which itself was not presented though. For instance, the presented words *apple*, *orange*, *kiwi*, *ripe*, *etc.* may potentially lead to the association of the critical lure *fruit*. An early recall was performed directly after presentation. The outcome measures were the number of correctly recalled words and the number of (falsely) reported critical lures. After the early recall, participants performed a recognition test. They were presented with a list of 40 words, 20 of which were previously presented and 20 of which were new, including the five critical lures. Here, based on the signal detection theory, we used as outcome the sensitivity index d´ in order to test how a participant was able to discriminate between old and new words. d´ was calculated using the formula d´ = Z(hit rate) – Z(false alarm rate) with hit rate defined as (hits/(hits + misses)) whereas hits were the correctly identified old words and misses were the old words that were not recognized (Macmillan and Creelman 1990). Respectively, the false alarm rate is the proportion of false alarms when a signal is absent (false alarms/(false alarms + correct negative) with false alarms being new words that were falsely identified as old words and correct negative being new words that are correctly identified as new words. The Z transformation was done using the statistical formula NORM.S.INV(hit) – NORM.S.INV(false alarm) in a Microsoft Excel spreadsheet. Perfect scores were adjusted using the formula 1–1/(2n) for perfect hits and 1/(2n) for zero false alarms, where *n* is the number of total hits or false alarms (20 and 20 respectively) (Haatveit et al. 2010; Macmillan and Creelman 1990). A higher d´ indicates that the signal can be detected more readily.

To assess *sustained attention*, the last test of the battery was the Psychomotor Vigilance Test (PVT), a reaction time task used to measure the speed of response to a visual stimulus (Davies and Parasuraman 1982). Participants responded to a counter that randomly appeared on screen with the reaction time displayed in msec. Average response time was used as outcome measure.

Moreover, *subjective affect* was evaluated with items from the Positive and Negative Affect Schedule (PANAS) (Krohne et al. 1996; Watson et al. 1988). The items “fatigue” (mean of drowsy, tired, sleepy and sluggish) and ‘serenity’ (mean of calm, relaxed, and at ease) were used as outcome measures.

Finally, *motivation* was measured with the mean of three visual analogue scales (VAS) that were filled after the working memory, the verbal memory, and the recognition task.

To assess *retention of information*, a delayed free recall test for the two sets of learned words was conducted by telephone 24 hours after the session. Participants were informed before discharge about the upcoming late recall task but were asked not to actively try to retain the words. Side effects were assessed regularly, before discharge, and also 24 h after the session.

### Statistical analysis

To control for within-subject effects, a linear mixed-effects model was conducted with the lmer function (lme4 package) in R, whereby subject identity was included as a random factor. The model further included a within-subject factor “placebo vs. stimulant” (MPH/MOD/CAF) and was performed for each outcome per stimulant type, respectively. Significance was established at the *p* < .05 level (two- tailed) and Benjamini-Hochberg (BH) correction for multiple comparisons for all 45 tests that were performed throughout the study was utilized, using a false discovery rate of 5% (Benjamini and Hochberg 1995). All values are reported as mean and standard deviation (SD) unless otherwise noted. All data was analyzed using IBM SPSS Statistics (version 23) and R programming language (version 3.5.1; R Core Team, 2018).

## Results

**Fig. 2:**
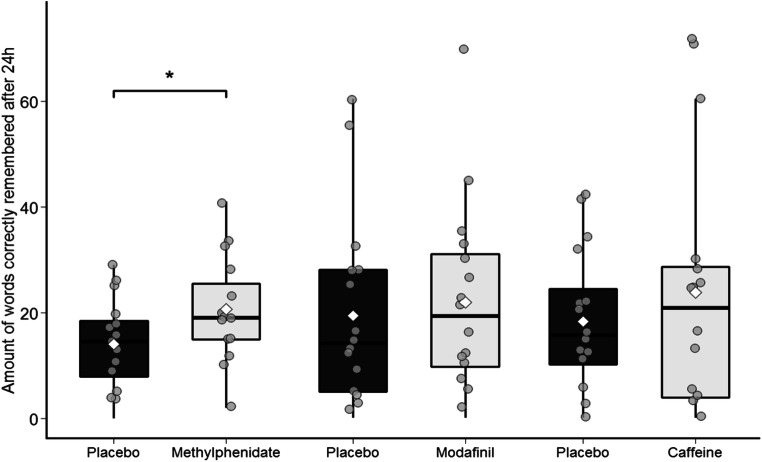
The amount of words remembered correctly 24 h after learning, per stimulant and their respective placebo condition. The grey dots represent the individual participants’ scores and the white diamond represents the group mean. *, *p* < .01

**Fig. 3:**
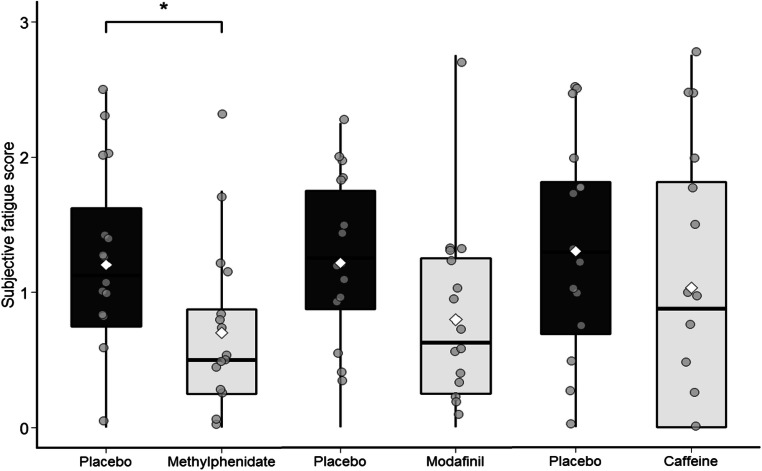
Subjective fatigue score per stimulant and their respective placebo condition. The grey dots represent the individual participants’ scores and the white diamond represents the group mean. *, *p* < .01

MPH led to significantly better late recall of the visually presented material (*F*(1,14.4) = 13.3, *p* = .003) 24 hours after learning (Fig. 2) but not to better retention of the audio material of the second declarative memory task that also included a false memory task (*F*(1,15) = 5.59, *p* = .032; not significant after BH correction for multiple comparisons). In a number of other tests including early recall of visual (*F*(1,14) = 5.88, *p* = .029) and audio (*F*(1,15) = 4.71, *p* = .047) material, implicit memory for audio material (*F*(1,15) = 6.44, *p* = .023), and creativity (*F*(1,15) = 5.82, *p* = .029), there was also a trend for better performance after MPH intake, but these results were not significant after BH correction for multiple comparisons. There was also no difference in performance in the tasks for working memory, logical reasoning, speed of processing, and sustained attention. In the subjective measurements, after receiving MPH, participants reported significantly less fatigue (*F*(1,15) = 15.5, *p* = .001; Fig. 3), whereas there was no difference in measures of motivation (*F*(1,15 ) = 5.43, *p* = .034; not significant after BH correction for multiple comparisons) and serenity.

There was no difference in the cognitive test battery between MOD and placebo, although there was a trend for less false memories in the early recall of the audio material (*F*(1,15) = 10.1, *p* = .006), which was however not significant after BH correction for multiple comparisons. In the subjective measures, MOD also did not differ from placebo, despite a trend to report less fatigue after MOD intake (*F*(1,15) = 6.36, *p* = .023; not significant after BH correction for multiple comparisons).

CAF, in comparison to placebo, led to better performance in the sustained attention task with significantly shorter reaction time (*F*(1,15) = 14.9, *p* = .002). There was a trend for better early recall of the audio material (*F*(1,15) = 4.81, *p* = .044; not significant after BH correction for multiple comparisons) and no difference in performance in the other tasks of the cognitive test battery or any of the subjective measures.

Since no differences between stimulant and placebo were found in the same cognitive test with two different stimulants, no differential comparison between drugs was performed on for multiple comparisons corrected data. However, in the uncorrected data, both MPH and CAF show to increase early recall of the audio material, a follow up analysis was performed to determine the differential effect between the substances on declarative memory performance. No significant differences between the two stimulants were found (*F*(1,30) = .51, *p* = .48). In addition, as less fatigue was reported in the uncorrected data after both MPH and MOD intake, a second follow-up analysis was performed to determine any differences between the stimulants on fatigue. Again, no significant differences between the two substances were found (*F*(1,30) = .06, *p* = .80).

The data was checked for outliers with a difference of at least three standard deviations from the mean and there were none. The results for each stimulant are presented in detail (with means and standard deviations for each outcome) in Tables 1, 2 and 3.

**Table 1:** Means and standard deviation (*SD)* of the outcome scores of the cognitive test battery and overview of all test values comparing the methylphenidate to the placebo condition

		Placebo		Methylphenidate		Methylphenidate vs Placebo	
	Test	Mean	*SD*	Mean	*SD*	*F*	*p*
Memory of visual material	Early recall—correct	31.00	13.33	38.67	14.95	F(1,14.6) = 6.06	.027
Logical reasoning	BOMAT—% correct	67.54	27.94	74.38	23.24	F(1,15) = 1.05	.322
Speed of processing	ZVT—sec	56.37	8.06	55.65	5.82	F(1,15) = 0.11	.741
Working memory	BackSpan	7.25	2.59	7.06	1.91	F(1,15) = 0.13	.718
Creativity	Alternate uses test Mean nr. of uses	10.75	3.57	12.04	0.32	F(1,15) = 5.82	.029
Memory of audio material	Early recall—correct	37.69	7.66	42.44	9.39	F(1,15) = 4.71	.047
False memory	Early recall—lures	1.13	0.89	1.00	0.89	F(1,15) = 0.21	.652
Implicit memory	d´	1.97	0.74	2.38	0.62	F(1,15) = 6.44	.023
Sustained attention	PVT; reaction time in ms	412	66.6	391	28.2	F(1,15) = 3.05	.101
Motivation	VAS— mean	6.02	2.47	6.93	1.95	F(1,15) = 5.43	.034
Subjective measures	Fatigue	1.2	0.7	0.7	0.6	F(1,15) = 15.5	.001*
	Serenity	2.8	0.8	2.6	0.8	F(1,15) = 0.87	.364
Late recall (24 h)	Visual material—correct	14.13	8.44	20.67	10.05	F(1,14.4) = 13.3	.003*
	Audio material—correct	24.13	10.4	30.63	12.48	F(1,15) = 5.59	.032
	Audio material—lures	1.62	1.15	1.13	1.03	F(1,14.4) = 2.31	.150

Marked with * are *p* values that were significant after using Benjamini-Hochberg correction for multiple comparisons, with a false discovery rate of 5%

**Table 2:** Means and standard deviation (SD) of the outcome scores of the cognitive test battery and overview of all test values comparing the modafinil to the placebo condition

		Placebo		Modafinil		Modafinil vs Placebo	
	Test	Mean	*SD*	Mean	*SD*	*F*	*p*
Memory of visual material	Early recall—correct	30.19	16.05	31.38	16.62	F(1,15) = 0.35	.564
Logical reasoning	BOMAT—% correct	71.20	20.19	65.52	20.80	F(1,15) = 0.85	.372
Speed of processing	ZVT—sec	56.59	10.71	55.88	10.75	F(1,15) = 0.22	.647
Working memory	BackSpan	7.75	1.13	7.63	1.67	F(1,15) = 0.06	.814
Creativity	Alternate uses test Mean nr. of uses	11.10	4.61	11.31	4.54	F(1,15) = 0.06	.810
Memory of audio material	Early recall—correct	38.56	9.95	39.50	8.11	F(1,15) = .24	.632
False memory	Early recall—lures	1.62	1.36	0.56	0.73	F(1,15) = 10.1	.006
Implicit memory	d´	2.17	0.53	2.25	0.84	F(1,15) = 0.12	.735
Sustained attention	PVT; reaction time in ms	387	34.9	375	33.1	F(1,15) = 2.82	.114
Motivation	VAS—mean	6.51	1.54	6.58	1.3	F(1,15) = 0.05	.821
Subjective measures	Fatigue	1.2	0.7	0.8	0.7	F(1,15) = 6.36	.023
	Serenity	2.7	0.8	2.6	0.7	F(1,15) = 0.03	.857
Late recall (24 h)	Visual material—correct	18.63	17.36	21.06	17.44	F(1,15) = 1.16	.299
	Audio material—correct	27.5	11.5	30.5	8.36	F(1,15) = 2.39	.143
	Audio material—lures	1.25	1.18	0.88	1.09	F(1,15) = 1.9	.188

Marked with * are *p* values that were significant after using Benjamini-Hochberg correction for multiple comparisons, with a false discovery rate of 5%

**Table 3:** Means and standard deviation (SD) of the outcome scores of the cognitive test battery and overview of all test values comparing the caffeine to the placebo condition

		Placebo		Caffeine		Caffeine vs Placebo	
	Test	Mean	*SD*	Mean	*SD*	*F*	*p*
Memory of visual material	Early recall—correct	33.88	21.22	35.81	22.53	F(1,15) = 0.58	.455
Logical reasoning	BOMAT—% correct	68.32	19.09	63.72	17.11	F(1,15) = 0.64	.435
Speed of processing	ZVT - sec	60.38	8.09	57.21	9.30	F(1,15) = 2.42	.140
Working memory	BackSpan	6.50	2.19	6.94	1.61	F(1,15) = 0.79	.387
Creativity	Alternate uses test Mean nr. of uses	11.35	4.50	11.48	3.44	F(1,15) = 0.03	.862
Memory of audio material	Early recall—correct	35.37	12.78	39.88	11.47	F(1,15) = 4.81	.044
False memory	Early recall—lures	1.38	1.26	1.19	0.98	F(1,15) = 0.25	.628
Implicit memory	d´	1.88	0.84	2.24	0.84	F(1,15) = 3.19	.094
Sustained attention	PVT; reaction time in ms	403	47.8	390	46.4	F(1,15) = 14.9	.002*
Motivation	VAS – mean	6.01	1.59	6.28	2.28	F(1,15) = 0.53	.477
Subjective measures	Fatigue	1.3	0.9	1.0	1.0	F(1,15) = 1.74	.208
	Serenity	2.7	0.6	2.8	0.7	F(1,15) = 0.52	.484
Late recall (24 h)	Visual material—correct	17.56	13.13	22.81	23.44	F(1,15) = 1.00	.333
	Audio material—correct	21.44	9.11	25.75	14.39	F(1,15) = 1.72	.209
	Audio material—lures	1.44	1.03	1.56	0.96	F(1,15) = 0.21	.652

Marked with * are *p* values that were significant after using Benjamini-Hochberg correction for multiple comparisons, with a false discovery rate of 5%

### Cardiovascular effects and side effects

All participants’ heart rate and blood pressure remained within normal range during the whole study. A total of 12 adverse events were reported, of which two events (headaches) were reported after placebo intake. Four events were reported after MPH intake (sleep-onset and sleep maintenance insomnia, and one participant reporting headache as well as restlessness). There were two cases of sleep-onset insomnia after MOD intake, whereas after CAF intake four adverse events were reported (restlessness, tiredness, and increased diuresis (reported twice)).

## Discussion

In this study methylphenidate, modafinil, and caffeine were compared with placebo in three different arms. After MPH intake, declarative memory performance was significantly better, an effect that was also shown previously both for the dose of 20 mg that was used in our trial and for a higher dose of 40 mg (Linssen et al. 2014; Linssen et al. 2012). In the previous studies, early recall was better after 20 mg and late recall was better after the higher dose. Here, we found an effect of 20 mg on 24 h late recall as well, an effect that has not been tested before. In our second declarative memory task, in which also a false memory task was integrated, no such effect was shown. This might have been due to cognitive overload, since two sets of more than 70 words each were presented within the same test battery. Besides, there were substantial differences between the two tests. The words of the first set were presented visually, while the words of the second set were presented auditorily and included a false memory task. However, no effect was found on implicit (false) memory nor on veridical memory in this task. Interestingly, there was no effect on the other cognitive tasks that were applied to test a variety of cognitive domains, including a test for sustained attention. The current results add to a body of data showing some effects of MPH on memory performance, while other cognitive domains remain unaffected (Repantis et al. 2010). This positive result is also in accordance with previously published studies with amphetamines (Ilieva et al. 2015). However, the magnitude of the positive effect, although statistically significant, was rather low, calling the actual utility of MPH as a memory enhancer into question.

In comparison to MPH, intake of 200 mg of MOD did not have a significant effect, although there was a trend for statistically significant better performance on the false memory task. The performance of the participants in the MOD arm was better in most tasks; this however failed to reach statistical significance in comparison to the placebo condition. The lack of significant effects can most likely be attributed to the small sample size since this pilot study was not adequately powered to detect small effects in non-sleep-deprived individuals. This is in line with the literature that shows positive effects of MOD mostly in sleep-deprived individuals (Repantis et al. 2010). However, newer studies have shown also positive results in some cases, especially on more complex tasks (Battleday and Brem 2015). It could however be that the neuropsychological test battery that was applied in this study was not able to capture more subtle effects of MOD. Given its wakefulness-enhancing properties, MOD most likely has more profound enhancing effects on sleep-deprived individuals, although it can be argued that this is not an enhancing effect per se, since sleep-deprivation does not represent the normal state.

Finally, CAF had a significantly positive effect on sustained attention, an effect that has repeatedly been reported in the past (Einöther and Giesbrecht 2013) and is being attributed to its antagonistic effect on adenosine receptors, in comparison to the mostly cathecholaminergic mediated effects of other stimulants. There is great variation in attention tasks that have been used in the literature while testing the effects of CAF, but there is general consensus that CAF improves basic aspects of attention as measured by reaction time tasks, such as the one used in our trial. Given that the sustained attention task, in which we observed effects, was the last task of the test battery, we can be sure that CAF was indeed still active until the end of the testing, despite having an earlier peak of maximal plasma concentrations than the other stimulants. Vice versa, we can be quite certain that the fact that CAF did not have any significant effect on any of the other cognitive domains that were tested earlier in the test battery is in accordance with the literature that shows that CAF has primarily a specific enhancing effect on attention.

The subjective effects of the three stimulants where also examined. Here, participants reported significantly less fatigue after MPH. There was also a positive trend for less fatigue after MOD intake; this however failed to reach significance after correction for multiple comparisons. CAF showed no subjective effects whatsoever, in contrast to a previous study testing caffeine as a beverage with the same test battery (Ullrich et al. 2015). This different level of subjective stimulation most likely represents the continuum of psychostimulant activation of the three stimulants tested here (Wood et al. 2014).

Taken together, our study showed specific but small effects for the different stimulants. In a post hoc analysis (provided in the supplementary material) to capture general cognitive enhancement effects in a larger sample, all stimulant arms were pooled together. We found significant results in favor of stimulant-intake compared with placebo in a number of tasks. These effects were however not significant after adding type of substance as between-subject factor. Hence, we can be sure that our experimental procedure was successful and that stimulants in total had positive effects on some cognitive processes. Moreover, this analysis indicated that our trial was underpowered to detect the rather small effects and especially the differential effects of each stimulant alone. Since no significant impact of two or more stimulants were observed on the same cognitive task, we were not able to explore differences between the drugs on these tasks, which could be attributed to the paucity of strong effects within each arm and stimulant. There are only a few studies that have examined in the same trial which prescription stimulant might be more effective and if so, in which cognitive domain. In a systematic review of placebo-controlled studies comparing MPH and MOD (against each other and placebo) on healthy non sleep-deprived individuals, we identified seven published articles that have dealt with this issue (search performed on the 19th of May 2020 with search terms used in a previous systematic review (Repantis et al. 2010), providing 24 results of which only seven fulfilled the predefined inclusion criteria). These seven articles reported results from four distinct trials, all of which were crossover trials. It was shown that MPH (20 mg) improved performance in a divided attention and a vigilance task, whereas MOD (200 mg) improved performance only in the vigilance task (Theunissen et al. 2009). In another study, increased alertness after MOD (400 mg) enhanced attentional performance (in this particular case: reduced spatial bias), whereas no such effect was shown for MPH (40 mg; Dodds et al. 2009). Both MPH and MOD enhanced perceptual processing speed only in a subgroup of participants with low performance in the placebo condition. This improvement correlated with subjective alertness and MOD also improved visual short-term memory storage capacities in the same subgroup (Finke et al. 2010). In a study that examined not only cognitive but also emotional effects, both MPH (60 mg) and MOD (600 mg) improved inhibitory performance (Schmidt et al. 2017b). MPH increased subjective anxiety and both MPH and MOD increased misclassification of emotions as anger in a facial emotion recognition task (Dolder et al. 2018; Schmidt et al. 2017a). On a neural level, for MOD, fear-induced activation in the middle and inferior gyrus correlated positively with subjective experienced feelings of fearfulness and depressiveness after MOD intake. Hence, the authors argued that the use of MPH and MOD for cognitive enhancement in these rather high doses might have potential adverse effects in emotion processing (Schmidt et al. 2017a). Finally, there has been one study comparing the two prescription stimulants not only to each other but also to CAF. This study applied each stimulant twice, the second dose 4 hours after the first and tested primarily the effects of stimulants on the performance of chess players. All substances increased reflection time while playing chess, leading to significantly more games lost on time. Thus, this more reflective decision-making led to worst performance under time pressure, an effect that was reversed when time constraints were not taken into account. In an extensive neuropsychological test battery that was also applied, only reaction time in a selective attention task (Stroop task) was significantly shorter after MPH. Finally, participants felt significantly less fatigued and reported more vigor after MPH and CAF (Franke et al. 2017).

Our results should be interpreted with caution since this pilot study has a number of limitations. First of all, it has a small sample size per arm; therefore we cannot exclude that small, substance-specific effects went undetected. With this trial, we are hoping to add to the literature that deals with the use of these substances as cognitive enhancers. Besides the social and ethical questions that are associated with the use of pharmacological substances for enhancement purposes (as we have argued elsewhere; Dresler et al. 2018), the risk-benefit assessment should take into account that individuals using stimulants for cognitive enhancement are not trying to treat debilitating symptoms but are looking to optimize an already good or “normal” performance. Therefore, it can be argued that the positive effects should be at least moderate in comparison to placebo in order to justify the possible risks. Therefore, although an underpowered study might be missing a small effect, it still adds an important information to the literature. Second, testing three stimulants simultaneously and examining their effects in diverse cognitive domains required correction for multiple comparisons, which may further contribute to the failure to detect some effects. In this study we were not interested in the effects on only one cognitive domain but aimed for an exploratory analysis of effects on different domains. That is why we did not formulate an a priori hypothesis on which stimulant would have which effect on each domain. Third, in order to avoid side effects and to examine effects comparable with those looked for and reported by persons using such substances for enhancement purposes in naturalistic settings, we used rather low to moderate doses; hence we might be underestimating the actual potential of the substances. Moreover, the doses might not be equipotent for the effect of each substance on each particular cognitive domain. It has been shown for instance that for sleep-deprived individuals, 200 mg of MOD had comparable effects with 600 mg CAF. However, in our case, in non-sleep-deprived individuals, we could show an effect of CAF on attention by applying only 200 mg, whereas 200 mg of MOD did not produce such an effect. This points to the interplay of baseline vigilance, substance, and cognitive domain in which an effect is looked for. It has been shown previously that stimulant effects on healthy individuals might depend on baseline performance as measured by intelligence scores and working memory capacity (Mehta et al. 2000; Randall et al. 2005). In our study, baseline characteristics did not have an impact on performance. However, our sample consisted mainly of individuals with high intelligence scores. It has been hypothesized that lower baseline performance might be linked to sub-optimal dopamine concentration within the prefrontal cortex and that stimulants possibly can alleviate this. On the other hand, persons with high baseline performance have already an optimal dopamine concentration and therefore do not benefit as much from stimulants, or in some cases performance might even decrease with increasing arousal (Wood et al. 2014), an effect that we did not see in our study either. Nevertheless, we still cannot rule out that domain-specific baseline differences might exist. Forth, we did not administer bodyweight-adapted doses nor measured individual substance plasma concentrations. Although the three substances have different peaks of maximal plasma concentrations (C_max_), all substances were applied 90 min prior to the cognitive testing. Given the different approximate times to reach C_max_ (MPH = 90 - 120 min; MOD = 120 – 240 min; CAF = 60 min), 90 min was chosen as a reasonable average to reach sufficient plasma concentration of each substance (Cappelletti et al. 2015; Dolder et al. 2018; Minzenberg and Carter 2008; Schmid et al. 2014; Swanson and Volkow 2003; Wood et al. 2014). Nevertheless, it is possible that our results do not represent the optimal performance that could be achieved with these substances. Besides, we cannot exclude that the fixed order of tests contributed to some extend to the lack of significant results, since not only concentration and therefore effect of the stimulant wears off with time but also cognitive capacities are depleted at the end of testing. By scheduling the testing always at the same time in the early afternoon, we tried to capture the effects of stimulants on afternoon tiredness, when performance is supposed to be at its lowest. The fact that the testing was performed at the weekend and that participants were asked to arrive at the laboratory well rested has probably alleviated afternoon tiredness though. Finally, it can be argued that since the participants left the laboratory after testing and late recall was assessed after 24 h by telephone, different environmental distractions across the two test days might have led to different encoding. In order to avoid this, participants were instructed to have a similar day routine on both days. As has been shown previously, this is a feasible testing method that avoids the burden of having the participants staying in the laboratory (Dresler et al. 2017).

To summarize, our study showed some moderate effects of two out of the three stimulants tested with MPH having positive effects on fatigue and long-term declarative memory and CAF-positive effects on sustained attention, whereas MOD had no significant effects in our trial. No trade-offs of negative effects on other cognitive domains were found. The results can direct further research that can focus specifically the effects of each stimulant on the cognitive domain that seems most promising, while possibly taking into account baseline performance in this particular domain as well.

## Supplementary information

ESM 1 (DOCX 21 kb)
