# Supplementary material for: Cognitive enhancement effects of stimulants: a randomized controlled trial testing methylphenidate, modafinil, and caffeine
Source: Psychopharmacology (Berl). 2020 Nov 17;238(2):441–51. doi: 10.1007/s00213-020-05691-w (PMC7826302; doi:10.1007/s00213-020-05691-w)
Supplement: Supplementary file 1 — (DOCX 21 kb) [file 213_2020_5691_MOESM1_ESM.docx]

Supplementary material

**Cognitive enhancement effects of stimulants:**

**a randomized controlled trial testing methylphenidate, modafinil and caffeine**

Dimitris Repantis*^1,2^, Leonore Bovy^3^, Kathrin Ohla^4^, Simone Kühn^2,5^, Martin Dresler^3^

Affiliations

1. Charité *–* Universitätsmedizin Berlin, corporate member of Freie Universität Berlin, Humboldt-Universität zu Berlin, and Berlin Institute of Health, Department of Psychiatry and Psychotherapy, Campus Benjamin Franklin, Berlin, Germany
2. Lise Meitner Group for Environmental Neuroscience, Max Planck Institute for Human Development, Berlin, Germany
3. Donders Institute for Brain, Cognition and Behaviour, Radboud University Medical Center Nijmegen, The Netherlands
4. Institute of Neuroscience and Medicine (INM-3), Jülich Research Centre, Germany
5. University Medical Center Hamburg-Eppendorf (UKE), Department of Psychiatry and Psychotherapy, Hamburg, Germany

**Supplementary analysis**: Besides the primary analysis, a post-hoc analysis was performed in which all stimulants were pooled together and tested against placebo. A linear mixed-effects model was conducted with the lmer function (lme4 package) in R, whereby subject identity was included as a random factor. In a further step, we included a within-subject factor “placebo vs. stimulant”. The analysis was performed for each outcome respectively. Significance was established at the p<.05 level (two- tailed) and Benjamini-Hochberg (BH) correction for multiple comparisons for all 15 tests that were performed was utilized, using a false discovery rate of 5% (Benjamini and Hochberg 1995). All values are reported as mean and standard deviation (SD) unless otherwise noted (Table S1). All data was analyzed using IBM SPSS Statistics (version 23) and R programming language (version 3.5.1; R Core Team, 2018).

In this analysis, there were significant results in favor of stimulant-intake in declarative memory (early and late recall), explicit verbal memory (early and late recall), false memory, implicit verbal memory, sustained attention and fatigue. However, there were no statistically significant results after adding type of substance as between-subject factor. Hence, we can be sure that our experimental procedure was successful and that stimulants in total had positive effects on some cognitive processes. Moreover, this analysis indicated that our trial was underpowered to detect the rather small effects and especially the differential effects of each stimulant alone.

**Table S1.** Means and standard deviation (*SD)* of the outcome scores of the cognitive test battery and overview of all test values comparing the stimulant to the placebo condition.

|  |  | | | **Placebo** | | | **Stimulant** | | | | **Stimulant vs**  **Placebo** | | |
| --- | --- | --- | --- | --- | --- | --- | --- | --- | --- | --- | --- | --- | --- |
|  | **Test** | | | **Mean** | | ***SD*** | **Mean** | | ***SD*** | ***F*** | | ***p*** |  |
| **Memory of visual material** | **early recall – correct** | | | 31.69 | | 16.89 | 35.21 | | 18.24 | F(1,46) = 5.33 | | .026* |  |
| **Logical reasoning** | **BOMAT – % correct** | | | 69.02 | | 22.32 | 67.87 | | 20.64 | F(1,47) = 0.1 | | .752 |  |
| **Speed of processing** | **ZVT - sec** | | | 57.78 | | 9.04 | 56.25 | | 8.71 | F(1,47) = 0.11 | | .169 |  |
| **Working memory** | **BackSpan** | | | 7.17 | | 2.09 | 7.21 | | 1.73 | F(1,47) = 0.02 | | .886 |  |
| **Creativity** | **alternate uses test**  **mean nr. of uses** | | | 11.07 | | 4.17 | 11.61 | | 3.75 | F(1,47) = 1.76 | | .191 |  |
| **Memory of**  **audio material** | **early recall - correct** | | | 37.21 | | 10.21 | 40.6 | | 9.64 | F(1,47) = 8.16 | | .006* |  |
| **False memory** | **early recall - lures** | | | 1.38 | | 1.18 | 0.92 | | 0.9 | F(1,47) = 5.39 | | .025* |  |
| **Implicit memory** | **d´** | | | 3.60 | | 2.01 | 2.29 | | 0.76 | F(1,47) = 6.13 | | .017* |  |
| **Sustained attention** | **PVT; reaction time in ms** | | | 401 | | 51.4 | 385 | | 36.7 | F(1,47) = 10.1 | | .003* |  |
| **Motivation** | **VAS – mean** | | | 6.18 | | 1.89 | 6.6 | | 1.87 | F(1,47) = 3.97 | | .052 |  |
| **Subjective measures** | **fatigue** | | | 1.24 | | 0.74 | 0.84 | | 0.79 | F(1,47) = 16.8 | | .0001* |  |
|  | **serenity** | | | 2.75 | | 0.75 | 2.69 | | 0.72 | F(1,47) = 0.21 | | .647 |  |
| **Late recall (24h)** | | **visual material – correct** | 16.77 | | 13.33 | | 21.53 | 17.61 | | F(1,46) = 5.32 | | .023* |  |
|  | | **audio material – correct** | 24.35 | | 10.47 | | 28.96 | 11.97 | | F(1,47) = 8.84 | | .005* |  |
|  | | **audio material – lures** | 1.44 | | 1.11 | | 1.19 | 1.05 | | F(1,47) = 2.17 | | .162 |  |

Marked with * are p-values that were significant after using Benjamini-Hochberg correction for multiple comparisons, with a false discovery rate of 5%.

**References**

Benjamini Y, Hochberg Y (1995) Controlling the false discovery rate: a practical and powerful approach to multiple testing. Journal of the Royal statistical society: series B (Methodological) 57: 289-300.
